# MR Imaging Findings in Alcoholic and Nonalcoholic Acute Wernicke's Encephalopathy: A Review

**DOI:** 10.1155/2014/503596

**Published:** 2014-06-24

**Authors:** Gaetana Manzo, Angela De Gennaro, Attilio Cozzolino, Antonietta Serino, Giacomo Fenza, Andrea Manto

**Affiliations:** ^1^Department of Biomorphological and Functional Sciences, University of Naples “Federico II,” Via Sergio Pansini 5, 80131 Naples, Italy; ^2^Department of Neuroradiology, Umberto I Hospital, Viale San Francesco 2, Nocera Inferiore, 84014 Salerno, Italy

## Abstract

Wernicke's encephalopathy (WE) is a severe neurological syndrome caused by thiamine (vitamin B1) deficiency and clinically characterized by the sudden onset of mental status changes, ocular abnormalities, and ataxia. Apart from chronic alcoholism, the most common cause of WE, a lot of other conditions causing malnutrition and decreasing thiamine absorption such as gastrointestinal surgical procedures and hyperemesis gravidarum must be considered as predisposing factors. Due to its low prevalence and clinical heterogeneity, WE is often misdiagnosed, leading to persistent dysfunctions and, in some cases, to death. Nowadays, MR imaging of the brain, showing T2 and FLAIR hyperintensities in typical (thalami, mammillary bodies, tectal plate, and periaqueductal area) and atypical areas (cerebellum, cranial nerve nuclei, and cerebral cortex), is surely the most important and effective tool in the diagnostic assessment of WE. The aim of this paper is to propose a state of the art of the role of MR imaging in the early diagnosis of this complex disease.

## 1. Introduction

Wernicke's encephalopathy (WE) is an uncommon but severe neurological syndrome, caused by thiamine (vitamin B1) deficiency. It is characterized by the sudden onset of altered consciousness, ophthalmoplegia, and ataxia [1]. This classic clinical triad, however, is present in only a minority of patients, making this condition often misdiagnosed and, consequently, life-threatening; its prognosis depends on prompt early intravenous administration of thiamine [2]. Magnetic resonance (MR) imaging, showing typical (thalami, mammillary bodies, tectal plate, and periaqueductal area) and atypical (cerebellum, cranial nerve nuclei, and cerebral cortex) signal-intensity alterations, is an essential tool to get the right diagnosis, especially when clinical presentation is incomplete [3]. The most common cause of thiamine deficiency is chronic alcohol abuse but a lot of other conditions such as gastrointestinal surgical procedures, hyperemesis gravidarum, and chemical therapy have been reported as predisposing factors [4].

We propose a review of the relevant literature focusing our attention on the role of MR imaging in the early diagnosis that, in recent years, has been fully confirmed.

## 2. Epidemiology

The prevalence of Wernicke's encephalopathy in the general population has been estimated from autopsy studies and varies from 0.4 to 2.8 [5, 6]; it seems to be much higher in alcoholics (AL) than in nonalcoholics (NA). These studies have revealed that the diagnosis of WE is often made only postmortem and less than 20% of the patients obtain a right diagnosis during life [6].

There is no racial predilection and the male sex is more affected than the female one (M : F ratio = 1.7 : 1) [7].

## 3. Aetiopathogenesis

Thiamine is a water-soluble vitamin involved in the maintenance of membrane integrity and osmotic gradients across cell membranes [8]. It is stored in body tissues, especially in the liver, predominantly as thiamine diphosphate (TDP) [9]. TDP plays an important role in the conversion of glucose into energy, acting as an essential cofactor for several enzymes in the Krebs cycle and in the pentose phosphate pathway. The Krebs cycle, also known as the tricarboxylic acid cycle (TCA cycle) or the citric acid cycle, is a crucial metabolic pathway that represents a key part of aerobic respiration in cells. It is constituted by a series of chemical reactions, occurring in the mitochondrion, that lead to the oxidation of acetate derived from carbohydrates, fatty acids, and amino acids into carbon dioxide, producing chemical energy in the form of adenosine triphosphate (ATP). In the citric acid cycle, TDP is an important coenzyme for the pyruvate dehydrogenate complex, which converts pyruvate, produced by glycolysis, into acetyl-CoA, and for the alpha-ketoglutarate dehydrogenase complex, that transforms alpha-ketoglutarate into succinil-CoA.

TDP also acts as cofactor of the enzyme transketolase, involved in the pentose phosphate pathway, a cytosolic process generating pentoses (5-carbon sugars), essential for nucleic acid synthesis [10]. A healthy adult requires approximately 1-2 mg of thiamine daily, depending on the carbohydrate intake. Body's reserves of thiamine are only 30–50 mg, so any malnutrition condition lasting more than 3-4 weeks can cause complete depletion of the vitamin's stores [3]. In case of thiamine deficiency, intracellular TDP is depleted leading to a series of metabolic alterations in the central nervous system. The decreased activities of the pyruvate dehydrogenate complex, the alpha-ketoglutarate dehydrogenase complex, and the transketolase, resulting in a reduction of Krebs cycle and pentose phosphate pathway efficiency, induce a cellular energy deficit due to reduced passage of pyruvate into the citric acid cycle, consequent intracellular accumulation of toxic intermediates such as lactate and alanine, reduction in PH, and cerebral lactic acidosis. Another effect is the intracellular accumulation of glutamate, derived from the transamination of alpha-ketoglutarate; glutamate limits the function of ATP-dependent cellular pumps, inducing failure to maintain cellular electrolyte homeostasis. Glutamate is consequently discharged in the extracellular space and, due to its function of excitatory neurotransmitter, neurons are exposed to excitotoxic damage. Excessive extracellular glutamate can bind to NMDA (N-methyl-D-aspartate) receptors, causing high calcium (Ca2+) concentration within the cells, leading to necrosis or apoptosis. Glial cells and neurons control of ionic gradients across the cell membrane is therefore impaired, causing cytotoxic edema. Thiamine deficiency also induces blood-brain barrier (BBB) dysfunctions with consequent vasogenic edema. Disruption of the BBB can be a result of physical processes such as the mechanical opening of endothelial tight junctions with increased vesicular transport across brain endothelial cells or of chemical-mediated alterations such as increased endothelial cell permeability due to the presence of amyloid precursor protein (APP) in susceptible brain areas. However, if thiamine is adequately administered before the occurrence of cellular death, the earliest alterations are reversible, creating the concept of “reversible biochemical lesion” [11]. Dysregulation of these thiamine dependent metabolic pathways represents the biochemical mechanism responsible for the signs and symptoms of WE.

The most common cause of thiamine deficiency is chronic alcohol abuse. Alcoholism is not directly responsible for vitamin B1 deficiency; its effects are related to the complications of liver cirrhosis such as problems to the gastrointestinal tract with low absorption rate at the mucosal level and consequent malnourishment [12]. Apart from alcohol, a lot of other conditions causing malnutrition and decreased thiamine absorption such as gastrointestinal surgical procedures (including gastric bypass surgery, gastrojejunostomy, gastrectomy, and colectomy) [13–18], therapy with intragastric balloon [19], hyperemesis gravidarum [20, 21], terminal tumor [22], chemical therapy [23, 24], allogenic stem cell transplantation [25], AIDS [26], anorexia nervosa [27], fasting [28], starvation [29], hemodialysis [30], pancreatitis [31], wrong formula feeding [32], parenteral nutrition, hyperalimentation [33, 34], and prolonged intravenous glucose infusion [35] have been reported as predisposing factors.

Besides Wernicke's encephalopathy, thiamine deficiency is the main cause of beriberi, a syndrome usually diagnosed in people whose diet consists mainly of polished white rice, which is very low in thiamine because the thiamin-bearing husk has been removed [36]. The clinical spectrum of this disease comprises cardiac beriberi, neuropathic beriberi, and gastrointestinal beriberi.

Cardiac beriberi is characterized by high-output cardiac failure with right-sided heart predominance [37]. Neuropathic beriberi is a painful sensorimotor peripheral neuropathy that typically affects the lower extremities [38]. In the recent literature, the term “dry beriberi” is usually used to describe neuropathic beriberi, while the term “wet beriberi” often refers to cardiac beriberi [39]. The idea of a primary gastrointestinal beriberi, characterized by abdominal pain, vomiting, and lactic acidosis, has been introduced recently but it might represent a consequence of cardiac beriberi [40].

## 4. Histologic Findings

In acute WE, pathologic findings comprise intra- and extracellular edema with swelling of astrocytes and oligodendrocytes and increased microglial cells, variable degrees of necrosis, demyelination, vascular proliferation, petechial hemorrhage, and disruption of brain-blood barrier [41–43]. These findings suggest that both vasogenic and cytotoxic edema are involved in the pathogenic mechanism of WE [8]; Liu et al. [44] suggested that vasogenic edema might precede cytotoxic edema. The most evident alterations are described at the level of the structures around the third ventricle such as the medial thalami, the periaqueductal grey matter, the mammillary bodies, and the tectal plate of the midbrain [41]. All these areas are considered typical sites of involvement. Because of their high oxidative metabolism, it has been suggested that these regions are particularly sensitive to thiamine deficiency [8]. The cerebellum, the dentate nuclei, the cranial nerve nuclei, the red nuclei, the caudate nuclei, the splenium, and the cerebral cortex are recognized as less commonly involved areas [3].

## 5. Clinical Manifestations and Diagnosis

Classically, WE is characterized by the sudden onset of a typical clinical triad: mental status changes, ophthalmoplegia, and ataxia; however, this complete triad can be seen in just one-third of patients. The most constant clinical finding is represented by mental status changes [45]. These changes comprise confusional state, spatial disorientation, dizziness, drowsiness, apathy, cognitive impairment with disturbance in memory and inability to concentrate, coma, and death; such symptoms derive from an involvement of thalamic nuclei or mammillary bodies [10, 46, 47].

Among ocular disorders, complete ophthalmoplegia occurs rarely, while the most common ocular abnormality is nystagmus, usually horizontal. Other ophthalmic alterations include bilateral decreased visual acuity, diplopia, palsy of both lateral recti or other ocular muscles and conjugate-gaze palsies resulting from lesions of the pontine tegmentum and of the abducens and oculomotor nuclei, torpid reaction of the pupils to light, retinal hemorrhage, papilledema, anisocoria, and ptosis [7, 10, 48, 49].

Equilibrium disorders comprehend gait ataxia, that can vary from mild gait disturbance to a complete inability to stand; it results from an involvement of cerebellar vermis and vestibular dysfunction. Some patients also experience polyneuropathy and dysarthria [7, 50].

The onset of the disease can be characterized by several other findings such as cardiac failure with hypotension and tachycardia, gastrointestinal symptoms like abdominal pain and nausea, hypothermia due to the involvement of the posterior hypothalamic regions, deafness due to thalamic involvement, and epileptic seizures in case of glutamatergic hyperactivity [51–55].

Because of this large variability in clinical presentation, in 1997, Caine et al. suggested that, in chronic alcoholics, a suspicion of Wernicke's encephalopathy should be based on two of the following four conditions: malnutrition, oculomotor abnormalities, cerebellar dysfunction, and an altered mental state [56]. In nonalcoholic patients, WE commonly presents with altered mental state without other symptoms and the diagnosis is often delayed or missed [4].

If left untreated, Wernicke's encephalopathy can lead to the Korsakoff syndrome, a form of memory disturbance characterized by anterograde and retrograde amnesia and confabulation, related to lesions in the thalamus and mammillary bodies [57].

The differential diagnosis should include stroke and intracranial hemorrhage, meningitis and encephalitis, brain tumors, cerebellar diseases, toxic ingestions, liver failure, Marchiafava-Bignami disease, and metronidazole-induced encephalopathy [58].

WE is essentially a clinical diagnosis; the determination of thiamine blood concentration and red blood cell transketolase activity can help to confirm it [59, 60]. However, these measurements are limited by a lack of specificity and technical difficulties. Computed tomography (CT) can show areas of reduced attenuation density at the level of the periaqueductal grey matter and the medial portion of the thalami but, in most cases, is negative in the acute phase of WE [3, 58]. Currently, MR imaging of the brain, with its high specificity, is surely the most important and effective tool to get the right diagnosis [61, 62].

## 6. MR Imaging Findings

**Figure 1:**
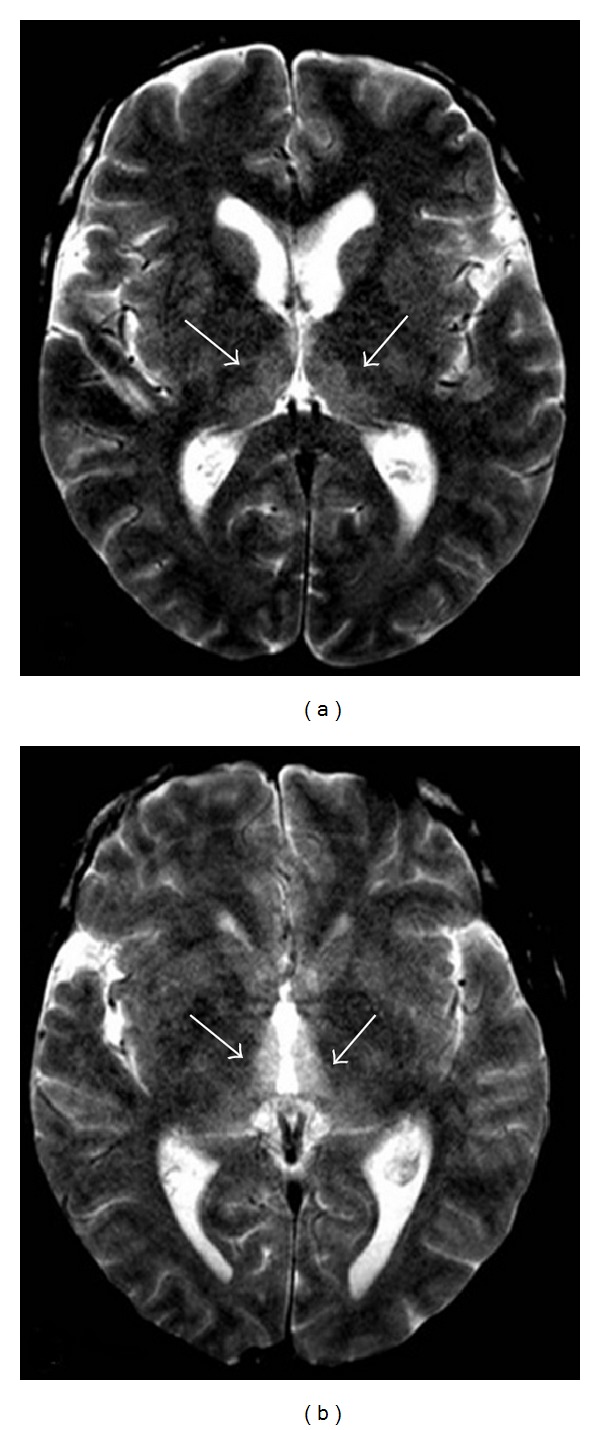
Forty-three-year-old man with a 10-year history of alcohol abuse. T2-weighted axial image showing bilateral and symmetric hyperintense signal alteration at the level of the medial portion of the thalami.

**Figure 2:**
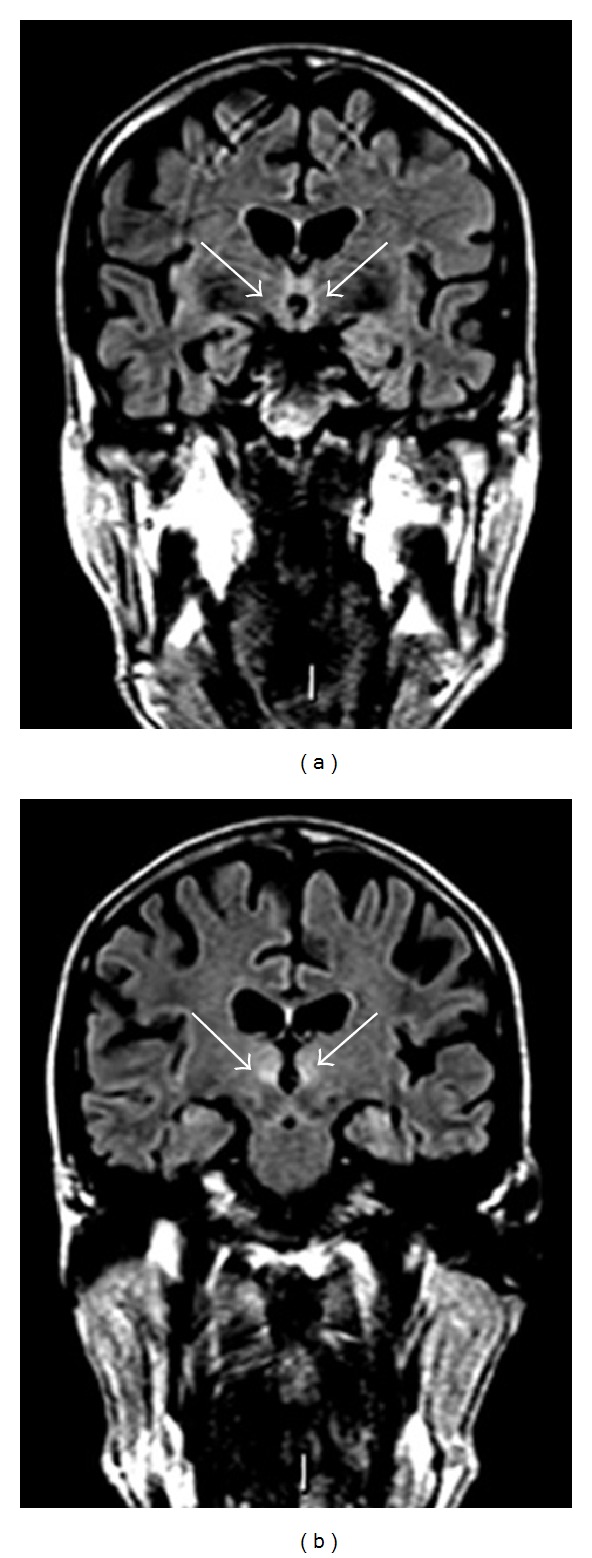
Fifty-year-old man with a 7-year history of alcohol abuse. FLAIR coronal images showing bilateral and symmetric hyperintense signal alteration at the level of the mammillary bodies (a) and the medial portion of the thalami (b).

**Figure 3:**
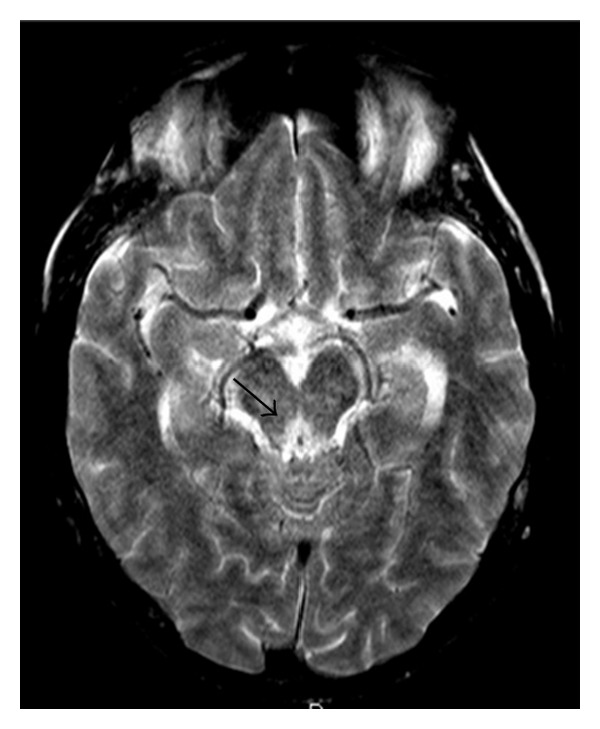
Sixty-four-year-old man with a 15-year history of alcohol abuse. T2-weighted axial image showing hyperintense signal alteration at the level of periaqueductal gray matter.

On MR imaging, the pathologic alterations described above are typically seen as bilateral and symmetrical T2w and FLAIR (Fluid Attenuation Inversion Recovery) hyperintensities in the thalami (Figure 1), mammillary bodies (Figure 2), tectal plate, and periaqueductal area (Figure 3) [3, 58, 63, 64]. In these areas, the maintenance of cellular osmotic gradients is considered to be strictly related to thiamine levels.

**Figure 4:**
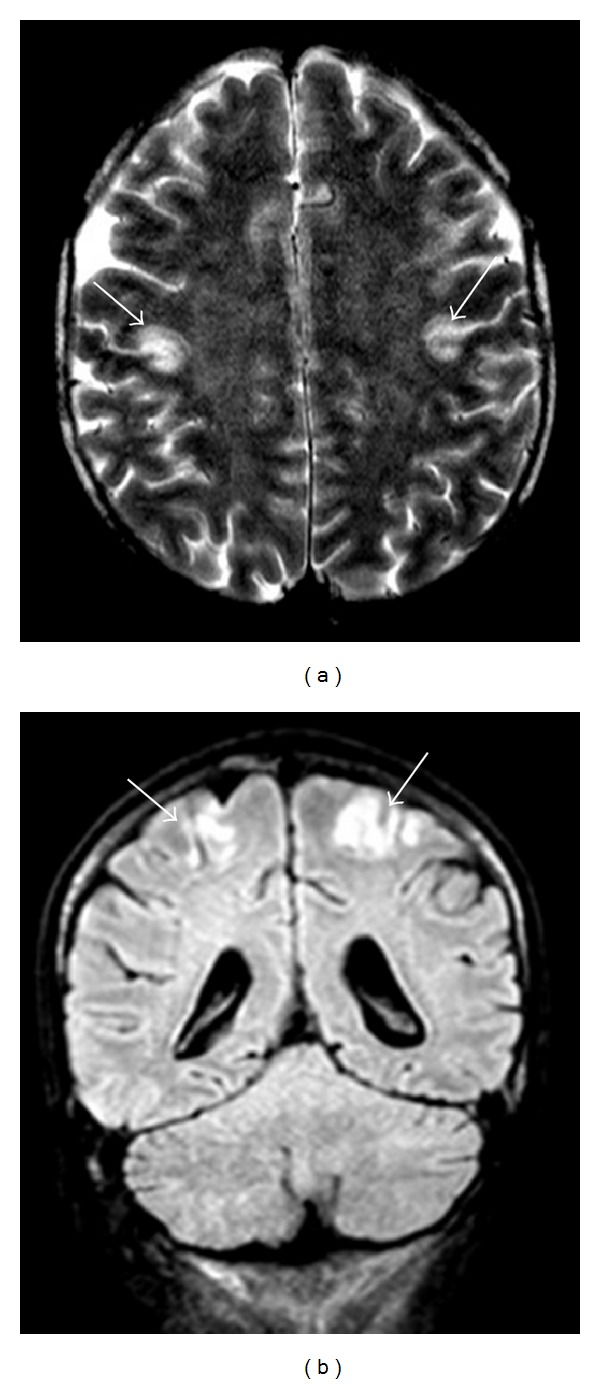
Forty-five-year-old man, affected by acute lymphoblastic leukemia, under chemical therapy, experiencing nausea and oral pain with consequent anorexia. T2-weighted axial (a) and FLAIR coronal (b) images showing bilateral hyperintense signal alteration at the level of the fronto-parietal cortex.

Signal intensity alterations in the dorsal medulla and the pons [65], cerebellar dentate nuclei, red nuclei [66], the substantia nigra of the midbrain [67], cranial nerve nuclei, the vermis and the paravermian regions of the cerebellum [68], the corpus callosum [69], the fornices [70], the head of the caudate nucleus [71], and the frontal-parietal cortex (Figure 4) [72] represent atypical MRI findings; they are almost always found in association with the typical findings.

Cerebellar signal intensity alterations can be observed in both AL and NA patients [73, 74]. Cerebellar involvement on imaging is rather rare but autopsy studies have demonstrated that the anterior-superior vermis or anterior hemisphere is affected in more than half of patients with WE [10]. The involvement of the caudate nuclei, in particular of the capita, may be due to their adjacent position to the lateral ventricles. Opdenakker et al. reported a case of hemorrhagic focus in the caput of the right nucleus caudatus of a patient with WE [75]. According to Zhong et al., the presence of lesions of the caudate nuclei, frequently observed in patients in comatose state, is a sign of pathologic evolution [76]. In accordance with the literature, cortical involvement indicates irreversible damage and poor prognosis [3, 4, 76]; recently, however, Cui et al. reported a case of nonalcoholic WE with cortical involvement in a patient that, after 45 days of vegetative state, regained consciousness thanks to parenteral thiamine administration [77].

The absence of MR signal-intensity alterations, however, does not exclude the diagnosis of WE. Gadolinium administration can be a useful tool to identify WE cases with negative MRI scan.

Contrast-enhanced T1-weighted images point out areas with disrupted blood-brain barrier and enhancement can be seen in about 50% of cases [43, 78, 79]. Strong enhancement of the mammillary bodies, for instance, can be the only sign of the disease [80, 81] and is more frequent in chronic alcoholics [2].

Important differences in MR imaging findings between AL and NA patients have been reported in the literature. First of all, in association with the typical alterations of WE, AL patients can also present atrophy of the mammillary bodies and the cerebellar vermis as a result of previous WE attacks. No atrophy, instead, is found in NA patients; in their case, signal intensity alterations represent the first result of thiamine-related metabolic pathway dysregulation [4, 41, 76].

Atypical findings, moreover, are found more frequently in NA patients. In 2009, Zuccoli et al. reviewed MR imaging findings and clinical records of fifty-six patients (24 AL and 32 NA) with WE and reported that signal-intensity alterations in areas considered atypical for the disease were noted only in the NA group and always in association with the typical findings; they explained these results speculating a possible protective effect of the alcohol on the brain areas that show atypical lesions in WE [82]. As an answer to this hypothesis, in 2010, Hygino da Cruz and his collaborators reported two cases of nonalcoholic WE demonstrating that both typical and atypical MR imaging findings may coexist [83]; in the same year, Sugai and Kikugawa added their experience describing atypical MRI findings in two patients with WE, both of whom were alcoholic [84]. In 2012, Ha et al. reported MR imaging findings of twenty-four patients (13 AL and 11 NA) with WE showing that the atypical MR imaging findings, including cerebral cortex and cranial nerve nuclei lesions, were present both in the AL group and in the NA group [85]. Few months later, Liou et al. described an interesting case of an AL patient affected by WE that showed lesions in olivary bodies, brain stem cranial nerve nuclei, and the dentate nuclei of cerebellum in absence of hyperintense lesions in the typically affected areas [86]. These findings suggest that further investigations are required before the relationship between alcohol and the brain lesions associated with WE can be understood.

In pediatric patients, signal-intensity alterations are often observed at the level of the basal ganglia with a characteristic involvement of the putamen, probably due to the high thiamine-dependent metabolism of these areas in children [87, 88].

As for the differential diagnosis on MR imaging, diseases showing symmetric signal-intensity alterations of the medial thalami should be considered: deep cerebral venous thrombosis [89, 90], paramedian thalamic syndrome [91], top-of-the-basilar syndrome (TOBS) [92], viral encephalitis [93], acute disseminated encephalomyelitis (ADEM) [94], atypical Creutzfeldt-Jakob disease [95, 96], primary cerebral lymphoma [97], influenza A virus infection [98], and West Nile virus meningoencephalitis [99]. All these diseases can be differentiated from WE thanks to their clinical characteristics and MR imaging findings; in particular, symmetric thalamic alterations in WE are usually associated with other characteristic neuroradiological signs such as symmetric alterations in the mammillary bodies, tectal plate, and periaqueductal area [63].

The differential diagnosis of symmetric signal-intensity alterations of the dentate nuclei, cranial nerves nuclei, abducens, red nuclei, and splenium should include metronidazole-induced encephalopathy (MIE) [3]. Metronidazole is believed to penetrate CSF and the central nervous system; it is safe but, at high dosages, can produce peripheral neuropathies and cerebellar dysfunction. At MR imaging, MIE can mimic WE presenting bilateral symmetric hyperintense lesions in cerebellar dentate nuclei, midbrain, dorsal pons, medulla, corpus callosum, and cerebral white matter; moreover, MIE can cause symptoms very similar to those produced by acute WE [100]. Metronidazole neurotoxicity may be mediated by the impairment of vitamin B1 action though its conversion to a thiamine analog and consequent vitamin B1 antagonism [101]. The differential diagnosis between metronidazole-induced encephalopathy and Wernicke's encephalopathy is even more complex, in patient with chronic gastrointestinal diseases under metronidazole treatment [82].

**Figure 5:**
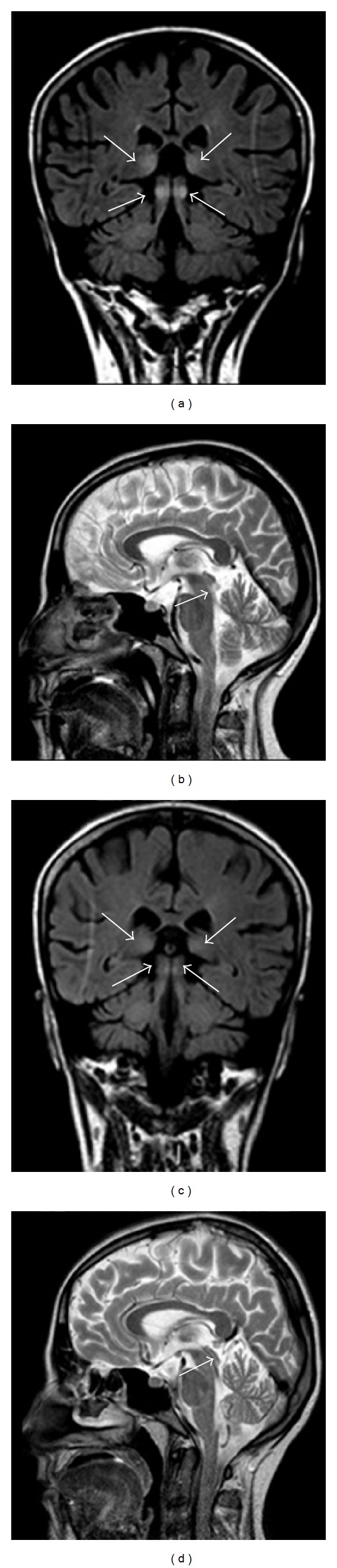
Thirty-three-year-old woman, primipara, in her 16th week of gestation, with a 3-week history of severe persistent nausea and vomiting accompanied by a 6 kg weight loss. FLAIR coronal (a) and T2-weighted sagittal (b) images showing bilateral and symmetric hyperintense signal alteration at the level of the medial portion of the thalami and of the lower portion of the tectal plate. A 3-week posttherapy (thiamine administered 100 mg/day intravenously for 10 days then 300 mg/day orally) brain MRI showed partial resolution of the signal abnormalities previously observed (c-d).

After an effective thiamine treatment, in conjunction with the resolution of clinical symptoms, MR imaging followup usually shows a gradual decrease and disappearance of signal-intensity alterations (Figure 5).

## 7. Role of DWI

**Figure 6:**
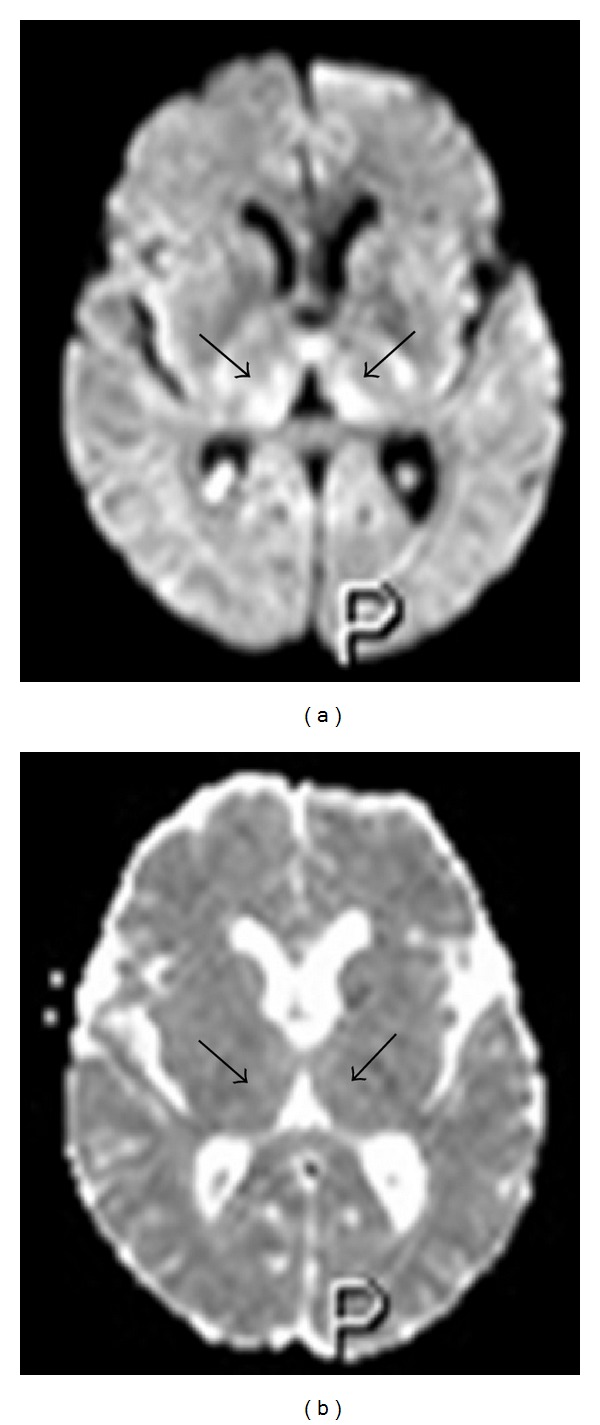
Fifty-three-year-old man in total parenteral nutrition. Axial diffusion-weighted image (a) showing bilateral and symmetric high signal at the level of the medial portion of the thalami with normal ADC values (b), indicating the presence of vasogenic edema.

**Figure 7:**
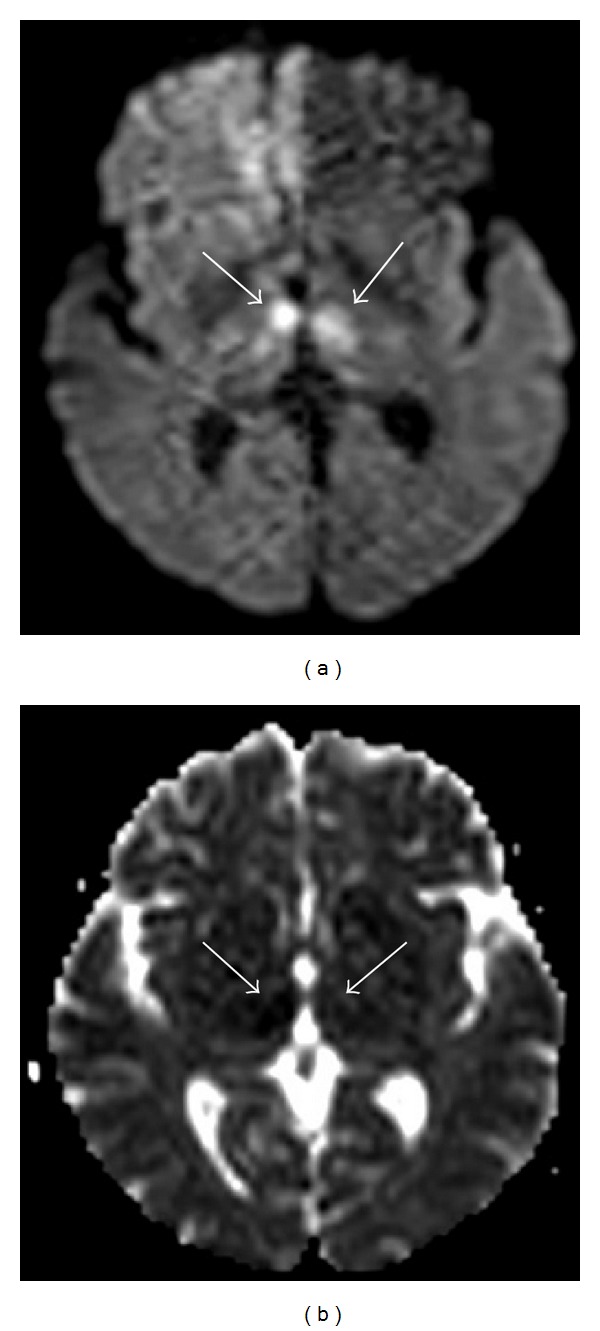
Forty-seven-year-old woman with a 16-year history of alcohol abuse. Axial diffusion-weighted image (a) showing bilateral and symmetric high signal at the level of the medial portion of the thalami with reduced ADC values (b), indicating the presence of cytotoxic edema.

In the diagnosis of acute Wernicke's encephalopathy DWI (diffusion-weighted imaging), detecting changes in water diffusion associated with cellular dysfunction is surely a valuable additional imaging sequence. The lesions can show hyperintensity on DWI images and reduced, normal, or increased ADC (apparent diffusion coefficient) values [102]. According to the literature, areas with high signal changes on DWI and normal or increased ADC values indicate the presence of vasogenic edema (Figure 6), characterized by elevated diffusion due to a relative increase in water in the extracellular compartment, whereas DWI hyperintensities with either decreased ADC represent cytotoxic edema (Figure 7) of the neurons and glial cells, characterized by restricted diffusion [103–110]. High signal intensity on DWI and decreased ADC values in acute WE do not always correspond to irreversible tissue damage; as discussed previously, the initial cellular alterations can be reversed so, when intravenous administration of thiamine is sufficient, signal-intensities abnormalities may regress and represent reversible cytotoxic edema, probably due to the osmotic dysregulation induced by decreased cellular energy levels. According to Chu et al., hyperintense signal alterations on DWI do not always indicate irreversibility but the presence of tissue at risk, similar to the cells in the ischemic penumbra [111]. This heterogeneity may result from disease severity, acuteness, and timing of imaging.

## 8. Role of MR Spectroscopy

**Figure 8:**
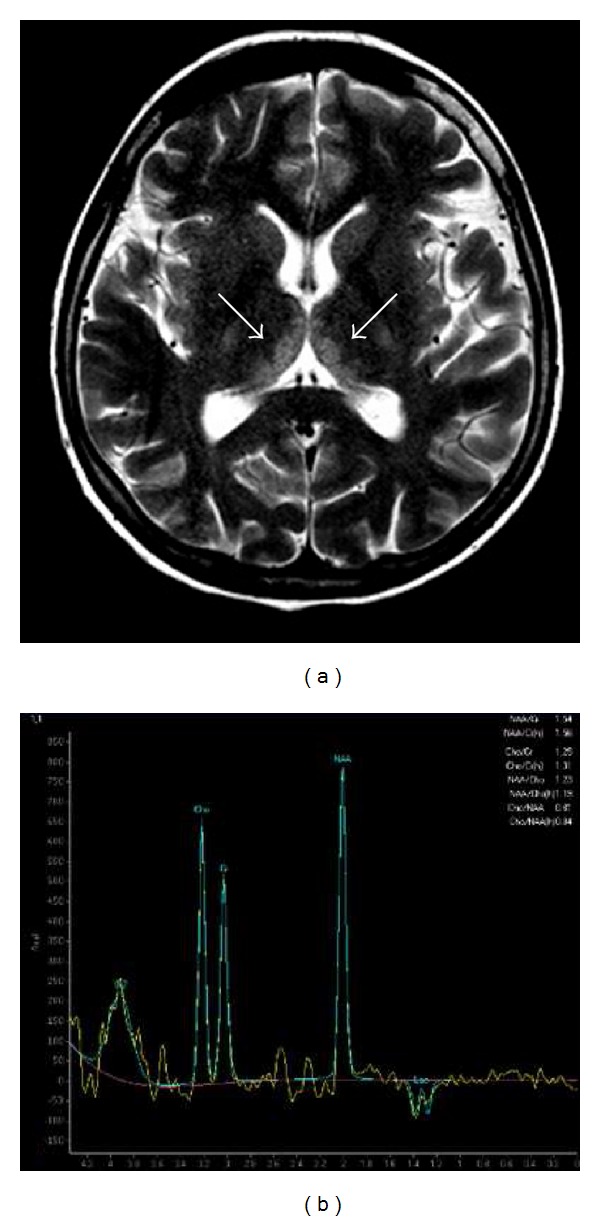
Forty-year-old man with a 10-year history of alcohol abuse. T2-weighted axial image (a) showing bilateral and symmetric hyperintense signal alteration at the level of the medial portion of the thalami and single-voxel MR spectroscopy image (b) demonstrating a decreased N-acetylaspartate (NAA)/creatine (Cr) ratio, a decreased N-acetylaspartate (NAA)/choline (Cho) ratio, and a lactate (Lac) peak, represented by a negative doublet (voxel taken from the right thalamus).

Proton MR spectroscopy is a powerful tool in the biochemical characterization of metabolic brain diseases in vivo. In the literature, there is a decent number of MR spectroscopy studies about thiamine deficiency but most of them have been performed on rats with very few studies applied to the investigation of WE in humans. In 2001, Murata et al. were the first to report proton MR spectroscopic findings in a patient with WE; they observed a decreased N-acetylaspartate (NAA)/creatine (Cr) ratio in the thalami and cerebellum and a lactate peak in the cerebellum (Figure 8). After thiamine administration, the NAA/Cr ratio increased at the level of the thalami but did not improve at the level of the cerebellum, suggesting irreversible tissue necrosis [112]. In 2002, Mascalchi et al. performed single-voxel MR spectroscopy in two patients with neurologic symptoms due to Wernicke's encephalopathy, before and after therapy, and placing their volume of interest in the thalami, in both cases, they observed a decreased NAA/Cr ratio without detectable lactate. They also reported an increased NAA/Cr ratio after thiamine therapy speculating it was presumably due to edema [113]. Few months later, in 2003, Rugilo and his collaborators reported proton MR spectroscopic findings in a patient with WE that showed a remarkable increase in lactate without decreased NAA/Cr ratio in the thalami. They hypothesized that the lactate peak was a result of the increased anaerobic oxidation of carbohydrates due to thiamine deficiency [114]. An analogous finding in a pediatric patient affected by WE has been recently reported by Rodan et al. Because of these few pieces of evidence, however, some other studies are required to assess the utility of MR spectroscopy as a potential diagnostic tool in Wernicke's encephalopathy [115].

## 9. Treatment and Prognosis

Wernicke's encephalopathy is a medical emergency and any therapeutic delay may result in permanent neurological damage or death. The treatment of suspected or manifest WE is based on the administration of thiamine. To date, there is still no consensus on its optimal dose, modality of administration, and treatment time. The traditional recommendation is a parenteral dosage of at least 100 mg of thiamine per day [116]; recently, some authors have recommended that patients should be given 200 mg of thiamine three times a day [117]. It should be given before or concomitantly with any carbohydrates because glucose can precipitate the disorder [118]. Duration of treatment also remains an enigma; it should be continued until there is no further improvement in signs and symptoms. Except for a small risk of anaphylactic reactions, the overall safety of intravenous thiamine is good [116, 118]. Initial improvements in acute symptoms can be observed within the first week but they usually take 1–3 months to resolve. Nevertheless, persistent neurologic dysfunctions, such as nystagmus and gait ataxia, are common. If not treated or inappropriately treated with low doses of thiamine, WE can lead to irreversible brain damage that can cause death in about 20% of cases or Korsakoff syndrome in 85% of survivors. AL patients are more predisposed to develop Korsakoff syndrome than NA patients, probably due to the possible occurrence of multiple nervous lesions induced by repeated episodes of thiamine deficiency in alcoholics [118].

## 10. Conclusions

Due to its low prevalence in the general population and clinical heterogeneity, Wernicke's encephalopathy is often misdiagnosed leading to disabling persistent dysfunctions and, in 20% of cases, to death. Chronic alcoholism is surely the most common cause of WE but it is important to remember that a lot of other conditions responsible for thiamine deficiency must be considered. The diagnosis of this syndrome is essentially clinical but the complete triad (changes in consciousness, ophthalmoplegia, and ataxia) can be seen in just one-third of patients. These problems, in association with the technical difficulties of determination of thiamine blood concentration, make MR imaging of the brain the most important and effective tool in the diagnostic assessment of WE. Bilateral and symmetrical T2w and FLAIR hyperintensities in typical (thalami, mamillary bodies, tectal plate, and periaqueductal area) and atypical (cerebellum, cranial nerve nuclei, and cerebral cortex) areas are characteristic. Contrast-enhanced MRI, pointing out areas with disrupted blood-brain barrier, is necessary to identify WE cases with negative scan. Signal-intensity alterations in atypical sites are found more frequently in NA patients but can be also detected in alcoholics. The role of DWI with ADC is still not well defined but may help to distinguish between vasogenic and cytotoxic edema. As to MR spectroscopy utility, further studies on its application to humans affected by WE are required. In conclusion, a responsible awareness of both clinical and neuroradiological Wernicke's encephalopathy features is fundamental to recognize it and reduce its morbidity and mortality.
